# Presence of Poly(A) Tails at the 3'-Termini of Some mRNAs of a Double-Stranded RNA Virus, Southern Rice Black-Streaked Dwarf Virus

**DOI:** 10.3390/v7041642

**Published:** 2015-03-31

**Authors:** Ming He, Ziqiong Jiang, Shuo Li, Peng He

**Affiliations:** 1 State Key Laboratory Breeding Base of Green Pesticide and Agricultural Bioengineering, Key Laboratory of Green Pesticide and Agricultural Bioengineering, Ministry of Education, Guizhou University, Guiyang 550025, China; E-Mail: hmher@126.com; 2 Plant Protection Station, Rural work office, Rongjiang County, Guizhou 557200, China; E-Mail: jiangzq_2015@126.com; 3 Institute of Plant Protection, Jiangsu Academy of Agricultural Sciences, Jiangsu Technical Service Center of Diagnosis and Detection for Plant Virus Diseases, Nanjing 210014, China

**Keywords:** *Southern rice black-streaked dwarf virus*, double-stranded RNA virus, poly(A) tails at mRNAs

## Abstract

*Southern rice black-streaked dwarf virus* (SRBSDV), a new member of the genus *Fijivirus*, is a double-stranded RNA virus known to lack poly(A) tails. We now showed that some of SRBSDV mRNAs were indeed polyadenylated at the 3' terminus in plant hosts, and investigated the nature of 3' poly(A) tails. The non-abundant presence of SRBSDV mRNAs bearing polyadenylate tails suggested that these viral RNA were subjected to polyadenylation-stimulated degradation. The discovery of poly(A) tails in different families of viruses implies potentially a wide occurrence of the polyadenylation-assisted RNA degradation in viruses.

## 1. Introduction

RNA of many eukaryotic viruses, ranging from DNA to RNA viruses, have 3' poly(A) tails [1], which are synthesized not only posttranscriptionally, but also by direct transcription from the poly(U) stretched template strand [2,3,4,5]. Regardless of synthesis mechanism used, the viral poly(A) tails have been considered to play crucial roles in RNA stability and translation, resembling roles of the stable poly(A) tails in eukaryotic mRNA [6,7]. Until recently, the function of poly(A) tails in destabilizing the viral RNA was revealed. The viral mRNA containing poly(A) or poly(A)-rich tails were detected in HeLa cells infected with *Vaccinia virus* (a double-stranded [ds] DNA virus) [8]. Furthermore, the polyadenylate tails were also found in *Tobacco mosaic virus* (TMV), *Cucumber mosaic virus* (CMV), *Odontoglossum ring-spot virus* (ORSV), *Cucumber green mottle mosaic virus* (CGMMV), *Tobacco rattle virus* (TRV), *Turnip crinkle virus* (TCV) and *Tobacco necrosis virus* (TNV) [9], seven positive-strand RNA viruses known to lack poly(A) tails and terminate 3'-termini with tRNA-like structure (TLS) or non-TLS heteropolymeric sequence [6]. The presence of poly(A) tails suggests that these viral RNAs are subjected to poly(A)-stimulated degradation. In this paper, the poly(A) and poly(A)-rich tails were first reported at the 3'-termini of the mRNAs of a dsRNA virus, Southern rice black-streaked dwarf virus (SRBSDV), generally recognized to lack poly(A) tails.

SRBSDV has been proposed as a new member in the genus *Fijivirus* of the family *Reoviridae* [10], which causes a serious rice disease in South China and Vietnam in recent years [11,12]. SRBSDV is most closely related to but distinct from *Rice black-streaked dwarf virus* (RBSDV), which is also a member of the *Fijivirus* genus [10,13]. SRBSDV genome contains 10 segments, named as S1-S10 in the descending order of molecular weight. Comparison of 10 genomic segments of SRBSDV with their counterparts in RBSDV suggests that SRBSDV encodes 13 open reading frames (ORFs) and possesses 6 putative structural proteins (P1, P2, P3, P4, P8, and P10) and 7 putative nonstructural proteins (P5-1, P5-2, P6, P7-1, P7-2, P9-1 and P9-2) [13]. At present, the functions of partial genes have been studied. The P6, encoded by S6, has been identified as an RNA silencing suppressor [14]. P7-1 induces the formation of tubules as vehicles for rapid spread of virions through basal lamina from midgut epithelium in its vector, the white-backed planthopper [15]. P9-1 is essential for viroplasm formation and viral replication in non-host insect cells and vector insects [16]. However, no reports are available to date to assign functions to the proteins encoded by other ORFs. The putative function of these proteins can only be postulated based on their RBSDV homologs. P1, P2, P3 and P4 are putative RNA-dependent RNA polymerase (RdRp), core protein, capping enzyme and outer-shell B-spike protein, respectively [13,17]. P8 and P10 are putative core and major outer capsid proteins, respectively [13,18]. SRBSDV mRNAs were considered to lack of poly(A) tails at the 3'-ends. However, in previous experiments, all 13 ORFs of the 10 RNA segments could be amplified via RT-PCR using oligo(dT)_18_ to prime cDNA synthesis as templates [19], suggesting that each SRBSDV mRNA might bear a potential poly(A) tail at the 3' terminus. In this paper, we confirmed that some of SRBSDV mRNAs were indeed polyadenylated at the 3' terminus in plant hosts.

## 2. Materials and Methods

### 2.1 Virus and RNA Extraction

SRBSDV isolate used in the experiment was obtained from rice and maize plants showing typical dwarf symptoms with white waxy galls in 2014 in 8 counties of 4 provinces in China, including Yunnan, Guizhou, Hunan, and Jiangxi provinces. Total RNA from infected rice and maize leaf and stem tissue were extracted following the standard protocol of TRIzol reagent (Invitrogen, Carlsbad, CA, USA). The isolate was identified as SRBSDV excluding RBSDV by reverse transcription RT-PCR using specific primers for distinguishing the two viruses [20].

### 2.2 Rapid Amplification of cDNA End (RACE) PCR

**Figure 1:**
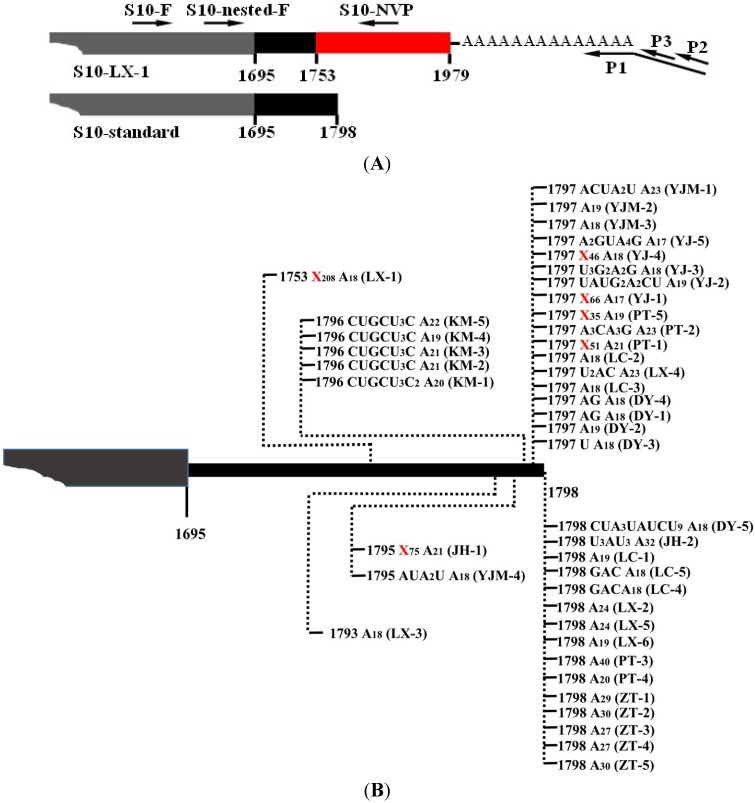
The 3' Rapid Amplification of cDNA End (RACE) detection of the polyadenylated *southern rice black-streaked dwarf virus* (SRBSDV) S10-mRNA. (**A**) Schematic diagram of the primers in S10-mRNA. The primers are displayed as arrowheads (Table 1), and the gray box, black box and red box indicate respectively partial ORF, 3' UTR and non-viral nucleotides in S10-mRNA. (**B**) Nature of 3' polyadenylate tails associated with S10-mRNA in plant. The poly(A) and poly(A)-rich tails of S10-mRNA are schematically presented, and vertical dashed lines with numbers indicate the exact positions of polyadenylate tails. Nucleotide compositions of the tails are shown, and long non-viral nucleotides are shown with X_n_. Isolate names in parentheses, DY: Duyun, PT: Pingtang, Guizhou province; JH: Jianghua, Hunan province; KM: Kunming, LC: Longchuan, YJ (YJM): Yingjiang, ZT: Zhaotong, Yunnan province; LX: Luxi, Jiangxi province. The number after abbreviation is the numbering of isolate clones. YJM isolates are from maize, and others are from rice.

To confirm characterization of the polyadenylate tails associated with viral mRNAs, the 3' Rapid Amplification of cDNA End (RACE) PCR was performed using BD SMART™ RACE cDNA Amplification Kit (TaKaRa, Dalian, Liaoning, China). In this case, reverse transcription reactions were performed using total RNA (respectively from infected rice and maize) as templates and adapter-oligo(dT) primer (P1) (Table 1) to prime first cDNA strand synthesis. 10 specific upstream primers and 10 nested primers respectively corresponding to SRBSDV each mRNA were designed according to China isolate HuNyy sequence information (GenBank No. JQ034348-JQ034357) (Table 1). Each of upstream primers was paired with adapter primer P2 (as downstream primer) for the 1st PCR amplification using PrimeSTAR HS DNA polymerase (TaKaRa) and cDNA as template. The PCR products from the 1st PCR reaction were subjected to a subsequent the 2nd PCR run with nested primers and adapter primer P3 (Figure 1A). The amplified products were analyzed by 1.5% agarose gel electrophoresis, and the resulting bands, in agreement with the predicted sizes, were individually cloned into pGEM-T Easy vector (Promega, Madison, USA) and subjected to sequence analysis. Approximately 5–10 clones from each isolate were randomly selected and sequenced.

**Table 1:** PCR primers used in the experiment.

Primer	Sequence (5'→3')	Target	Reference GenBank No.
S1-F	TCAGTGCTCAAGGCTCACAAGATTGAAG	S1-mRNA	JQ034348
S1-nested-F	ATTCATGAACTTAATGGGCGCAGAGTG		
S2-F	CGGCACATCTTCACCCGCAGACTTC	S2-mRNA	JQ034349
S2-nested-F	CTGATGAATTGCTCGACCGTTACATTAG		
S3-F	GATGGGATTAGCGAAATTGCATTTGGAG	S3-mRNA	JQ034350
S3-nested-F	TGCATGGACATTCATTTTCAGATCAAG		
S4-F	TAGATTTTGTTATTCCCGGTGTTCGAGAAG	S4-mRNA	JQ034351
S4-nested-F	AGTGCGGATGTGGCTGCAGATAAATTC		
S5-F	TGTGATCAGTGCCATGTCCACTAGCATC	S5-mRNA	JQ034352
S5-nested-F	AATCATCCCTGTGCGCTTCGACTTAG		
S6-F	CGATACTCTGATGAAACAGGCGAAGCTC	S6-mRNA	JQ034353
S6-nested-F	TGAGAACCAATGGAGCGCGTATGGA		
S7-F	ACTACTTCAGCTGAAGATGTCGACGCAC	S7-mRNA	JQ034354
S7-nested-F	TTGGCAAGCGATGGAAAGAAGATGG		
S8-F	CGTATTGGACGATGAGCGCAACTTTG	S8-mRNA	JQ034355
S8-nested-F	TGAATTAGCGTTCGTACCTCATTCGCTG		
S9-F	TTGGACTTGGCTAACTACGTTCGACAAC	S9-mRNA	JQ034356
S9-nested-F	GGAATTGGATGATCGAGTTGAAAAATTGG		
S10-F	CTCCCTGCATCGATTACATCAAACTTGG	S10-mRNA	JQ034357
S10-nested-F	GCCAACAATTTATTGAAGGCGGATCG		
S10-NVP	TTCCATCTCTATCATTCAGTCAAG	S10-mRNA	
Adapter-oligo(dT) (P1)	GCTGTCAACGATACGCTACGTAACGGCATGACAGTG(T)_18_VN	Poly(A) tails	
Adapter primer P2	GCTGTCAACGATACGCTACGTAACG	Adapter	
Adapter primer P3	CGCTACGTAACGGCATGACAGTG	Adapter	

## 3. Results and Discussion

**Figure 2:**
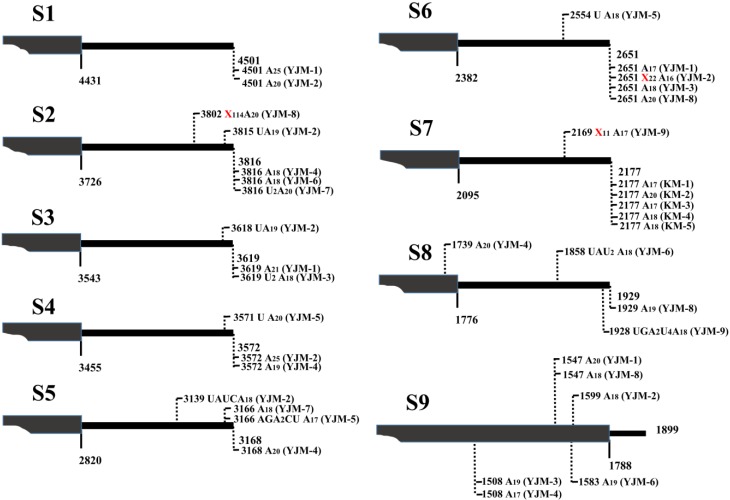
Nature of 3' polyadenylate tails associated with SRBSDV S1-S9 mRNAs in plant. The poly(A) and poly(A)-rich tails of S1-S9 mRNAs are schematically presented respectively, and vertical dashed lines with numbers indicate the exact positions of polyadenylate tails. Nucleotide compositions of the tails are shown, and long non-viral nucleotides are shown with X_n_. Isolate names in parentheses, KM: Kunming (from rice), YJM: Yingjiang (from maize), Yunnan province. The number after abbreviation is the numbering of isolate clones.

After 3' RACE, the 3'-termini sequences of viral mRNAs were obtained, and the results indicated that SRBSDV mRNAs indeed possessed ploy(A) or poly(A)-rich tails in plant hosts. Taking S10-mRNA as an example to analyze the nature of poly(A) and poly(A)-rich tails, a total of 42 polyadenylated viral mRNA molecules were cloned from rice and maize plants. In addition to 10 mRNAs bearing poly(A) tails exclusively comprised of adenosines, a large number of mRNAs possessed poly(A)-rich tails (Figure 1B). Notably, the heterogeneity of these poly(A)-rich tails was confined to their 5' ends, and they all terminated in homogenous adenosines (17–23 nt) (Figure 1B), which was possibly due to the 3' bias of oligo(dT)-dependent reverse transcription. Most poly(A)-rich tails were not at the downstream of S10-mRNA entire 3' untranslated region (UTR), and replaced partial 3' UTR sequences. For example, the tail of isolate LX-1 replaced 3' UTR sequence of S10-mRNA from the nucleotide 1753 (Figure 1B). In some poly(A)-rich tails (isolate JH-1, LX-1, PT-1, PT-5, YJ-1 and YJ-4), there were more non-viral nucleotides (35–208 nt) preceded polyadenylates, which was considered to originate from host plants. In order to further certify the presence of poly(A) tails and exclude non-specificity of reverse transcription reaction, these non-viral nucleotides was used to design downstream primers (e.g., S10-NVP) to perform PCR with upstream primer from S10 (Figure 1A), and the result of amplification was positive (data no shown), indicating sufficiently the existence of mRNA bearing ployadenylate tails. Moreover, poly(A) or poly(A)-rich tails were also discovered at the 3'-ends of viral S1-S9 mRNAs (Figure 2). All amplified products based on 3' RACE were weak (data no shown), implying that a small fraction of SRBSDV mRNAs was polyadenylated.

To our knowledge, dsRNA viruses are lack of poly(A) tails at the 3'-ends of the genome segments and their mRNAs. Interestingly, in this paper, we demonstrated that some viral mRNA molecules were polyadenylated at their 3'-terminus in plant cells infected with SRBSDV (a dsRNA virus). Besides their crucial roles for mRNA stability and translation efficiency, the polyadenylate tails were recently described as involved in viral RNA degradation [8]. The Poly(A)-stimulated RNA degradation occurs throughout the prokaryotic and eukaryotic cells [21,22,23,24,25,26]. Generally, the degradation process comprises three sequential steps: endonucleolytic cleavage, addition of polyadenylate tails to the cleavage products, and exonucleolytic degradation [21,26,27]. The transient poly(A) or poly(A)-rich stretches can act as landing sites to recruit 3'-5' exoribonucleases for further degradation [21,22,26,27], which might be one of ancestral roles of polyadenylation. This evolutionarily conserved mechanism has been confirmed to play critical roles in rapidly removing redundant RNAs in cells, thereby maintaining the stability of gene expression [26,28,29].

In this study, the non-abundant presence of SRBSDV mRNAs bearing polyadenylate tails was considered to represent degradation intermediates of an RNA decay pathway, rather than to convey protection to mRNAs. Recently, a dsDNA virus, *Vaccinia virus*, was linked with the conserved RNA degradation mechanism, and non-abundant, fragmented viral mRNAs bearing poly(A) or poly(A)-rich tails were detected in human cells infected with this virus [8]. Such polyadenylation-stimulated RNA degradation was also found in seven positive-strand RNA viruses from distinct virus families and genera known to lack poly(A) tails [9]. The discovery of poly(A) tails in three different types of viruses (positive-strand RNA virus, dsDNA and dsRNA virus) implies potentially a wide occurrence of the polyadenylation-assisted RNA degradation in viruses, which might represent a yet-unknown interaction between virus and host.
